# Survival prognostic factors in patients with acute myeloid leukemia using machine learning techniques

**DOI:** 10.1371/journal.pone.0254976

**Published:** 2021-07-21

**Authors:** Keyvan Karami, Mahboubeh Akbari, Mohammad-Taher Moradi, Bijan Soleymani, Hossein Fallahi

**Affiliations:** 1 Medical Biology Research Center, Kermanshah University of Medical Sciences, Kermanshah, Iran; 2 Department of Animal Science, Ferdowsi University of Mashhad, Mashhad, Iran; 3 Department of Statistics, Ferdowsi University of Mashhad, Mashhad, Iran; 4 Department of Biology, School of Sciences, Razi University, Kermanshah, Iran; University of Kentucky, UNITED STATES

## Abstract

This paper identifies prognosis factors for survival in patients with acute myeloid leukemia (AML) using machine learning techniques. We have integrated machine learning with feature selection methods and have compared their performances to identify the most suitable factors in assessing the survival of AML patients. Here, six data mining algorithms including Decision Tree, Random Forrest, Logistic Regression, Naive Bayes, W-Bayes Net, and Gradient Boosted Tree (GBT) are employed for the detection model and implemented using the common data mining tool RapidMiner and open-source R package. To improve the predictive ability of our model, a set of features were selected by employing multiple feature selection methods. The accuracy of classification was obtained using 10-fold cross-validation for the various combinations of the feature selection methods and machine learning algorithms. The performance of the models was assessed by various measurement indexes including accuracy, kappa, sensitivity, specificity, positive predictive value, negative predictive value, and area under the ROC curve (AUC). Our results showed that GBT with an accuracy of 85.17%, AUC of 0.930, and the feature selection via the Relief algorithm has the best performance in predicting the survival rate of AML patients.

## Introduction

Acute myeloid leukemia (AML) is a clonal disorder that is associated with a reduction of differentiation of the myeloid lineage, accumulation of immature progenitors in the bone marrow, resulting in hematopoietic failure [1]. The peripheral blood is the most involved organ in this disease, while infiltration of other organs such as the brain and/or the lung is uncommon and found mostly in cases with high blast counts in the blood [2].

The criterion for AML according to World Health Organization (WHO) is observing at least 20% myeloblasts in the marrow (or blood) with myeloid lineage [3]. Besides the ≥20% criterion are cases of core-binding factor (CBF)-AML, nucleophosmin 1 (NPM1)-mutated AML, or acute promyelocytic leukemia (APL); in each of them, the AML diagnosis is blast %-independent. Cases with more than 20% blasts without markers are mentioned as acute undifferentiated leukemia (AUL) and mostly treated like AML [2].

Multiple studies have suggested the contribution of both genetic factors and clinical variables in predicting overall survival (OS) and event-free survival (EFS). Basically, besides aging, which is considered as an independent prognostic factor, the genetic mutation of RUNX1, ASXL1, and TP53 are associated with poor prognosis and a lower chance of survival. TP53 mutation and complex karyotype give independent prognostic information and their combination results in the worst outcome [4]. Generally, it has been suggested that 75% of variations are related to genomic instability, and the other 25% are associated with clinical, treatment, and demographic variables. So far, 37 Models with a combination of all of these factors could predict remission or life expectancy only in 75% to 80% of cases [4]. This emphasizes the need to find other prognostic factors with higher accuracy.

Machine learning (ML) techniques have become a popular tool for the prediction of disease outcomes. For example, it can identify existent patterns and relationships between datapoint to predict the outcome of cancer [5]. Machine learning is a type of artificial intelligence that mostly develops computer programs and could accommodate new data whenever they are available [6]. So, computer models developed based on the previous data could be used for classification, prediction, and detection processes. Among ML techniques, feature selection-based techniques are selecting a subset of features from the original set of features [7]. These techniques are very convenient to use and relatively accurate to implement for prediction processes.

Numerous studies have used prediction models for the prediction of cancer survivability. For example, Delen et al. used artificial neural networks (ANN), Decision Trees (DT), and logistic regression (LR) methods in breast cancer patients [8]. Also, Chao et al. have used several models such as support vector machine (SVM), LR, and a C5.0 decision tree model to the prediction of breast cancer survival [9]. Another study introduced a different method for the prediction of survival of breast cancer patients. They used the Naive Bayes (NB), Trees Random Forest (TRF), 1-Nearest Neighbor (1NN), AdaBoost (AD), Support Vector Machine (SVM), RBF Network (RBFN), and Multilayer Perceptron (MLP) machine learning techniques alongside with 10-cross fold technique for the prediction of breast cancer survival [10]. Similar work was conducted by Delen et al. for prostate cancer patients using SVM, DT, ANN, and LR [11]. Interestingly, in most clinical studies Random Forest (RF) appears to be the most preferred algorithm [12, 13].

In the current study, we have applied multiple machine learning techniques to a dataset of AML to evaluate the predictive power of these techniques in the prediction of survival outcomes (alive or death) of the patients. The aim is to improve the subsequent therapy and management of patients for increasing survivability. So, the application of machine learning models for accurate prediction of the survival in patients with acute myeloid leukemia based on clinical data was assessed in our study.

## Materials and methods

Several variables have been registered for patients in the current database including numerical and categorical variables. The patients were classified according to the French-American-British (FAB) system. The data were prepared for analysis using data mining tools and algorithms. Feature selection was done via feature weighting methods and after this section, 25 of the high weight features were selected to continue the analysis. As listed in the following sections, multiple classifiers were trained and evaluated for their ability to predict the survival of patients.

### Dataset

Data used in this study were obtained from the Leukemia Sample Bank at the University of Texas M. D. Anderson Cancer Center that were collected between January 15, 1998, to March 9, 2006. The primary dataset contained information regarding 249 patients with AML. Several variables have been evaluated for patients. These variables show in Tables 1 and 2 including categorical and some numerical variables that were used in the study. A list of protein features used in the analysis can also be found in the (S1 Table). The missing values in categorical variables and numerical features replaced by the mode and average of the missing value in each class as imported into the RapidMiner software (RapidMiner7.5.003, www.rapidminer.com) [14].

**Table 1:** Statistical description of categorical variables in the AML patients.

Variables	Description	Value	Frequency (%)
**SEX**	Patients’ gender status	Female	115(46.2)
		Male	134(53.8)
**RACE**	Ethnicity	Asian	3(1.2)
		Black	21(8.4)
		Hispanic	29(11.6)
		White	196(78.7)
**ZUBROD.S**	Zubrod score runs from 0 to 5, with 0 denoting perfect health and 5 deaths.	0–2	236(94.8)
		3–4	13(5.2)
**INFECTION**	People with AML can get infections that don’t seem to go away or may get one infection after another.	No	176(70.7)
		Yes	73(29.3)
**PRIOR _MAL**	History of malignant tumor	No	190(76.3)
		Yes	59(23.7)
**PRIOR _XRT**	History of XRT	No	228(91.6)
		Yes	21(8.4)
**PRIOR _ CHEMO**	History of Chemotherapy	No	220(88.4)
		Yes	29(11.6)
**AHD**	Antecedent hematological disorder 0–120.	≥ 50	11(4.4)
		< 50	238(95.6)
	Defined as a heme disorder noted at least 2 months before the leukemia diagnosis. There are three fields, yes/no, a second with the # of months it was noted beforehand and a third that specifies what the heme abnormality was.		
**FAB**	French-American-British classification	M0	17(6.8)
		M1	33(13.3)
		M2	71(28.5)
		M4	74(29.7)
		M4EOS	9(3.6)
		M5	6(2.4)
		M5A	13(5.2)
		M5B	9(3.6)
		M6	7(2.8)
		M7	5(2)
		RAEBT	5(2)
**CG.group**	Cytogenetic Group classification	Favorable	21(8.4)
		Intermediate	115(46.2)
		Unfavorable	113(45.4)
**D835**	FLT3 mutation at amino acid 835	Negative	233(93.6)
		Positive	16(6.4)
**ITD**	Internal tandem duplication	Negative	205(82.3)
		Positive	44(17.7)
**VITAL STATUS**	The survival status of the patients	Alive	78(31.3)
		Dead	171(68.7)

**Table 2:** Statistical description of numerical variables in the AML patients.

Variables	Description	Min	Max	Mean	Std. Deviation
**Age**	Age at enrollment	16.1	87.2	60.12	16.29
**CR_duration (Months)**	Duration of the complete remission	0.9	250.1	50.75	47.75
**ALBUMIN**	a protein made by liver	1.5	4.9	3.34	0.70
**BILIRUBIN**	a brownish yellow substance found in bile	0.1	6.2	0.65	0.58
**CREATININE**	a chemical compound left over from energy-producing processes in muscles	0	6	1.14	0.65
**FIBRINOGEN**	a protein produced by liver	0	980	432.73	158.59
**LDH**	Lactate dehydrogenase	10	15544	1907.98	2097.79
**WBC**	White blood cell	0.2	373	41.55	54.87
**CD 7**	Monoclonal antibody Leu-9	0	99	17.03	25.44
**CD10**	a cancer specific antigen	0	91	3.56	7.55
**CD13**	a cancer specific antigen	1.5	100	75.73	25.82
**CD19**	a cancer specific antigen	0	98	6.64	15.05
**CD20**	a cancer specific antigen	0	65.8	1.59	4.82
**CD33**	a cancer specific antigen	0.4	100	82.45	25.02
**CD34**	a cancer specific antigen	0	99.9	46.32	39.45
**PLT**	Platelet count	4	511	72.96	75.31
**PM Blast**	Percent Bone Marrow blasts	0	98	58.37	23.47
**PB_Blast**	Percent Peripheral blood blasts	0	99	40.67	30
**HGB**	Hemoglobin	3.5	26.4	9.73	1.91

### Data cleaning

The preprocess of cleaning and formatting of the data often is crucial for obtaining a good fit of the model and better predictive ability. Therefore, correlated attributes with a Pearson correlation coefficient greater than 0.95 were removed from the list. Additionally, numerical attributes with a standard deviation less than or equal to a given threshold (0.1) were assumed to be useless in the analysis and removed from the initial dataset. The remaining data treated as the processed dataset and was used for conducting this study [15].

### Feature selection

The performance of many algorithms would decrease by using irrelevant features. So, the selection of relevant features is an important step during data mining. Here, the feature selection was conducted through feature weighting approaches including information gain, information gain ratio, Gini index, chi-squared, correlation, relief, uncertainty [16]. We then selected 25 features with the highest weighting score for the following model evaluation steps (So we have used more than 30% of all features analyzed). In the following, we give a short description of the feature selection algorithms used in methods.

1. Information gain
  Information gain is an entropy-based feature evaluation method, widely used in the field of machine learning. As information gain is used in feature selection, it is defined as the amount of information provided by the feature items for the text category. Information gain is calculated by how much of a term can be used for the classification of information, to measure the importance of lexical items for the classification [17, 18].
2. Information gain ratio
  The information gain ratio for splitting according to some feature "A" is the gain divided by the entropy of A. One advantage of the gain ratio is that the gain ratio less prefers a finer partition. This measure is proposed by Jia et al. [19].
3. Gini index
  Gini-Index is a split measure of total variance across the K classes. This rule is used for splitting attributes in choosing the most appropriate splitting attribute at each node in a decision tree [20, 21].
4. Chi-squared
  The Chi-square test is a statistical technique used in statistics to test the independence of two events. In feature selection, the two events are the occurrence of the term and the occurrence of the class.
5. Correlation
  Correlation is a statistical method of the linear relationship between two variables. Features with high correlation are more linearly dependent and hence have almost the same effect on the dependent variable. So, when two features have a high correlation, we can drop one of the two features.
6. Relief
  Relief is considered one of the most successful feature selection algorithms that estimate the quality of features according to how well their values distinguish between the instances of the same and different classes that are near each other. Relief was originally defined for two-class problems and was later extended (ReliefF) to handle noise and multiclass datasets [22, 23].
7. Uncertainty
  Symmetric uncertainty has been obtained by normalizing Mutual Information to the entropies of two variables. This nonlinear measurement indicates the correlation between two variables. In feature selection, this measure evaluates an attribute by measuring its symmetrical uncertainty concerning the class [24].

### Model evaluation

Based on what was reported in the literature, six machine learning techniques were used to study the data including RF, DT, LR, Naive Bayes, W-Bayes Net, and GBT. We performed 10-fold cross-validation for all datasets. In each fold, the mining models are fitted to the training data and the test observations are used for validation. The conventional machine learning algorithms were assessed by their accuracy, kappa, sensitivity, specificity, positive predictive value, negative predictive value, and AUC (area under the ROC curve) which were used to investigate the performance of the machine learning algorithms. The ROC is a curve generated by plotting the true positive rate (TPR) against the false positive rate (FPR) at various threshold settings while the AUC is the area under the ROC curve. In summary, a model with good predictive should have an AUC closer to 1 (1 is ideal) than 0.5. Some definitions concerning the measurements are given to explain how the indicators are gained. The following abbreviations were used for empirical quantities: P (# positive samples), N (# negative samples), TP (# true positives), TN (# true negatives), FP (# false positives), FN (# false negatives). Accuracy was estimated using the ratio of (TP+TN)/(P+N). Positive predictive value (PPV) was estimated by TP/(TP+FP). Negative predictive value (NPV) was estimated by TN/(TN+FN). Sensitivity was estimated by TP/P. Specificity was estimated by TN/N. Accuracy was used to select the optimal model using the largest value. Sensitivity in this context is also referred to as the true positive rate or Recall, and PPV is also referred to as precision. In the following, a short description of the data mining algorithms used in model selection are given:

1. Random Forest
  A random forest is a classifier consisting of a certain number of random trees, specified by the number of trees parameter for classification, regression and other tasks. RF will create multiple classification and regression (CART) trees, each trained on a bootstrap sample of the original training data and searches across a randomly selected subset of input variables to determine the split [25–27]. Random decision forests correct for decision trees’ habit of overfitting to their training set [28]. As for this study, the default number of trees (ntree = 500) in RF was employed to assess the model accuracy.
2. Decision tree
  Decision trees are the most popular learning method for data mining. Decision trees come closest to meeting the requirements for serving as an off-the-shelf procedure for data mining. Because They are relatively fast to construct and produce interpretable models. They are invariant under scaling and various other transformations of feature values, are robust to the inclusion of irrelevant features, and produce inspectable models [28].
3. Logistic regression
  Logistic regression is a technique borrowed by machine learning from the field of statistics. This method is appropriate to conduct a regression analysis with a dichotomous (binary) dependent variable. The logistic regression model is very popular due to its simplicity and ability to make inferential statements about model terms [29, 30].
4. Naive Bayes
  The Naive Bayes algorithm is a predictive model and classification technique based on the Bayes theorem. It simplifies the probabilities of the predictor values by assuming that all of the predictors are independent of the others. Naive Bayes Classifier is one of the simple and most effective classification algorithms which can be computed quickly and performs competitively in many cases [30].
5. Bayes Net
  Bayesian networks, also known as belief networks (or Bayes nets for short) are a probabilistic graphical structure that represent a set of variables and their probabilistic relationships via a directed acyclic graph. Bayes nets are ideal for taking an event that occurred and predicting the likelihood that any one of several possible known causes was the contributing factor. For example, a Bayesian network could represent the probabilistic relationships between diseases and symptoms. Given symptoms, the network can be used to compute the probabilities of the presence of various diseases [31, 32].
6. Gradient Boosted Tree
  The Gradient Boosted Trees Operator trains a model by iteratively improving a single tree model. After each iteration step, the Examples are reweighted based on their previous prediction. The final model is a weighted sum of all created models. Training parameters are optimized based on the gradient of the function described by the errors made. Gradient boosting of regression trees produces competitive, highly robust, interpretable procedures for both regression and classification [33].

## Results

### Patient characteristics

The mean age of the 249 patients included in the study was 60.12±16.29 years. Description of categorical and some numerical variables are summarized in Tables 1 and 2, respectively. Table 1 shows the frequencies and the percentages of the categorical variables in each class. Table 2 reports the minimum (Min), maximum (Max), mean, and standard deviation (Std. Deviation) of the numerical variables.

### Feature selection

**Fig 1:**
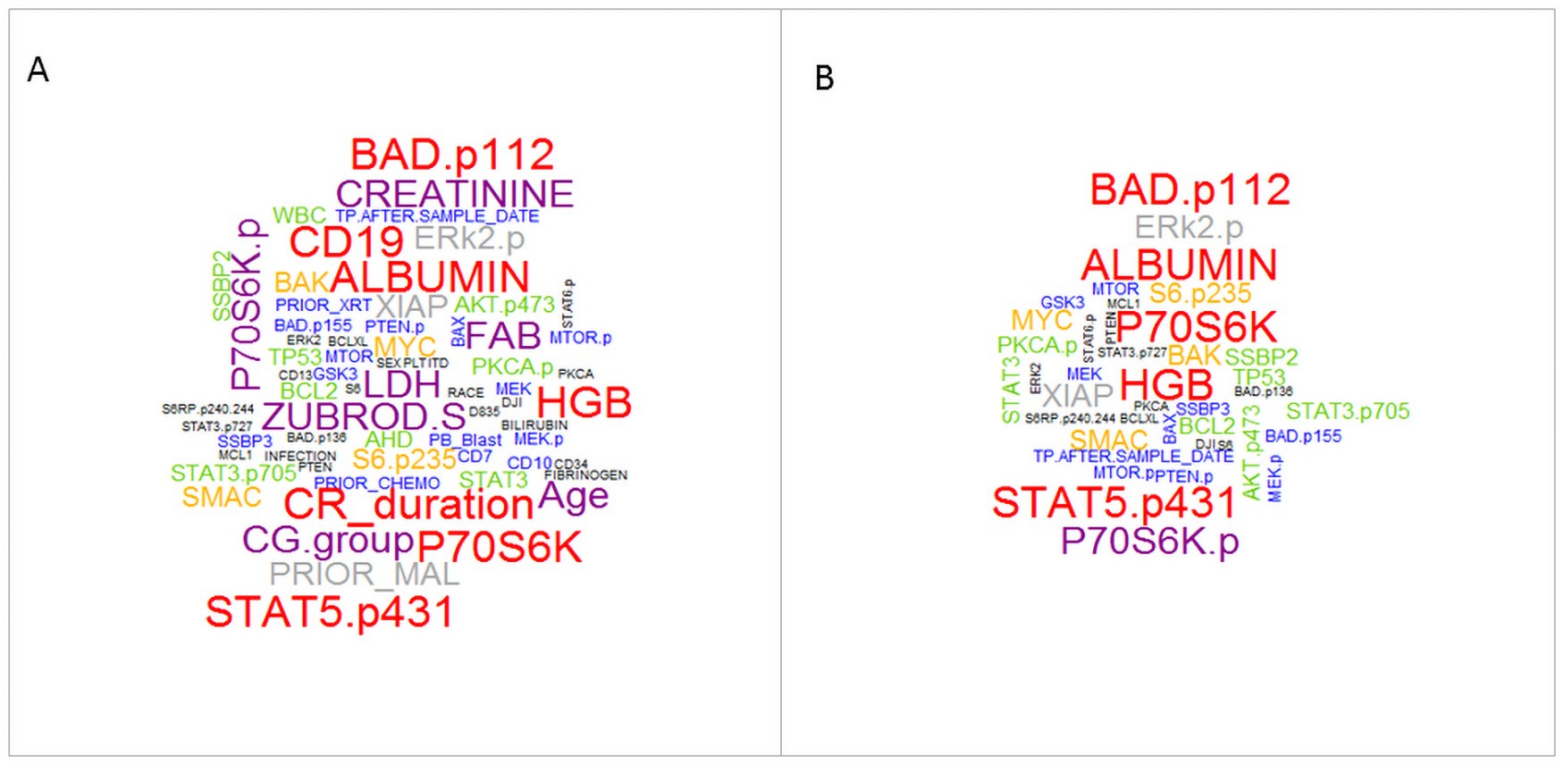
The most important features (A) and the most important proteins (B) in all feature selection algorithms.

**Fig 2:**
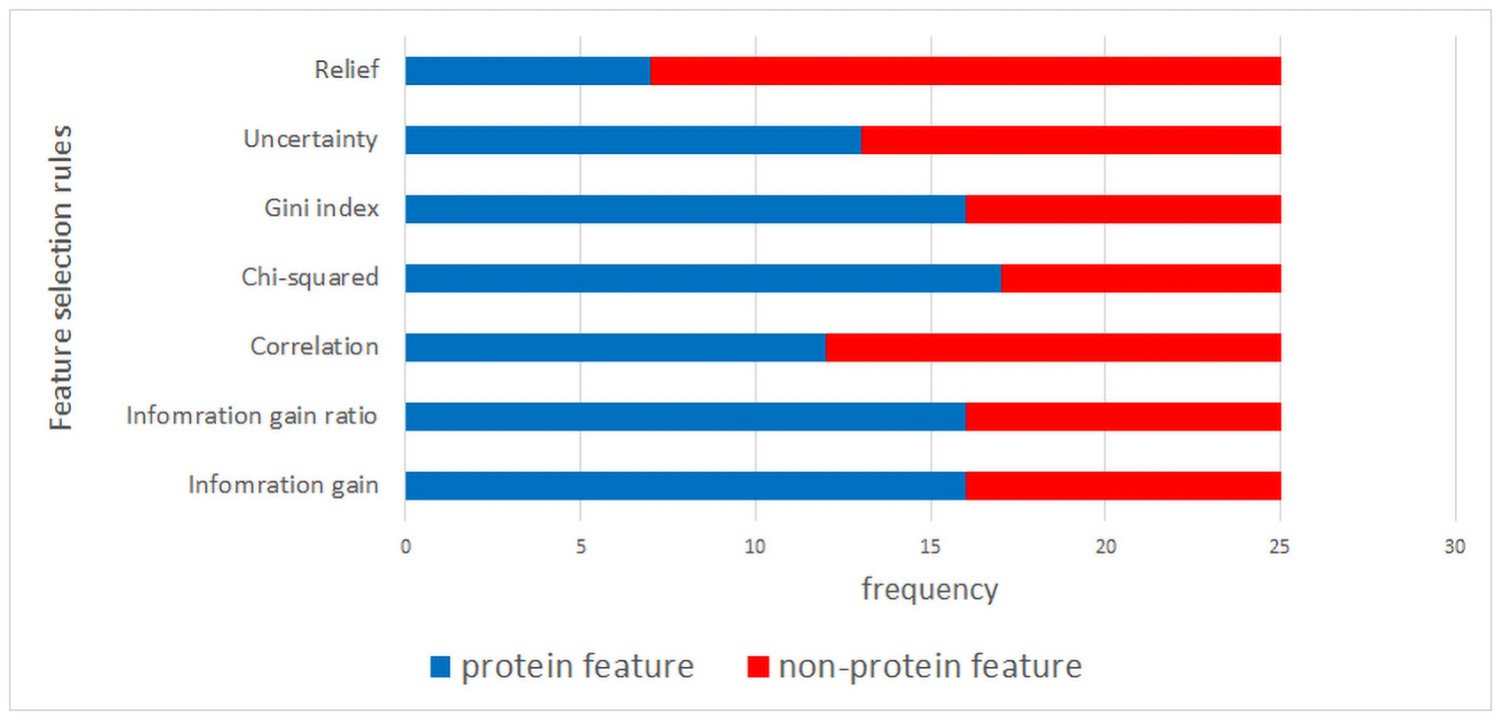
Distribution of protein features among studied datasets.

The 25 features (about 30% of all features) with the highest weighting score were selected using various feature selection operators and are presented in Table 3. A visual representation of all features selected by feature selection techniques is shown in Fig 1A, which includes the most important feature that had a higher weight score in multiple feature selection algorithms. Also, the most important protein features among all feature selection algorithms are given in Fig 1B. The size of each word shows the importance of that term, as it appears more frequently in all feature selection techniques. According to the figures, the most frequent non-protein features are CD19, CR_duration while more frequent protein features include Albumin, HGB, STAT5.p431, BAD.p112, P70S6K. Fig 2 presents the contribution of the protein and non-protein features for each dataset produced by various feature selection algorithms.

**Table 3:** Top 25 most important features selected by various feature selection methods.

Method	Features
**Information gain**	CR_duration, Age, FAB, CG.group, LDH, CREATININE, CD19, P53, PRIOR_MAL, P70S6K.p, AHD, BAD.p112, ALBUMIN, ZUBROD.S, HGB, XIAP, ERk2.p, P70S6K, MEK, STAT5.p431, STAT3, STAT3.p705, BCL2, SMAC, BAK.
**Chi-squared statistic**	CR_duration, Age, CG.group, STAT5.p431, FAB, XIAP, ALBUMIN, BAD.p112, CD19, CREATININE, ERk2.p, P70S6K.p, ZUBROD.S, MYC, P70S6K, LDH, STAT3.p727, AKT.p473, BAX, SSBP2, S6.p235, WBC, PKCA.p, MTOR, HGB.
**Gini index**	CR_duration, Age, CG.group, FAB, LDH, CREATININE, CD19, XIAP, P70S6K.p, ZUBROD.S, HGB, ERk2.p, STAT5.p431, P70S6K, BAD.p112, PRIOR_MAL, TP53, AHD, BCL2, ALBUMIN, STAT3, MEK, SMAC, BAK, SSBP2.
**Information Gain Ratio**	CR_duration, ALBUMIN, MCL1, SSBP3, WBC, HGB, BAD.p112, GSK3, MTOR, S6.p235, STAT5.p431, STAT6.p, BILIRUBIN, CREATININE, CD10, CD13, CD19, CD7, PB_Blast, AKT.p473, BAD.p155, ERk2.p, MEK.p, MTOR.p, MYC.
**Relief**	CR_duration, CG.group, Age, TP.AFTER.SAMPLE_DATE, CD19, SEX, RACE, PRIOR_MAL, PRIOR_XRT, FIBRINOGEN, PRIOR_CHEMO, PKCA.p, ALBUMIN, CD34, HGB, PTEN.p, FAB, PKCA, STAT5.p431, ITD, LDH, INFECTION, ZUBROD.S, P70S6K, PB_Blast.
**Uncertainty**	CR_duration, CG.group, Age, CD19, CREATININE, WBC, LDH, FAB, PRIOR_MAL, CD10, ZUBROD.S, XIAP, STAT5.p431, ALBUMIN, BAD.p112, MYC, P70S6K.p, HGB, BAX, P70S6K, ERk2.p, CD7, PLT, S6.p235, S6.
**Correlation**	CR_duration, Age, CG.group, PRIOR_MAL, CREATININE, ZUBROD.S, HGB, BAK, CD19, D835, FAB, PRIOR_CHEMO, TP53, BCL2, SMAC, LDH, ALBUMIN, TP.AFTER.SAMPLE_DATE, P70S6K.p, P70S6K, XIAP, PRIOR_XRT, STAT3.p705, BAD.p112, AHD.

### Assessing the predictive ability of the model

In this section, we evaluate the ability of the selected models to predict the survival status of AML patients. Table 4 gives the accuracy percent of the proposed prediction models using a 10-fold cross-validation procedure over various feature selection algorithms. Table 5 reports the Accuracy, Kappa value, Specificity, Sensitivity, PPV (precision), NPV, and AUC of the proposed prediction models.

**Table 4:** Accuracy (%) of the prediction algorithms using a 10-fold cross-validation procedure for each of the datasets.

Criterion	RF	DT	LR	Naive Bayes	W-Bayes Net	GBT
**Information Gain**	84.33	81.52	82.30	81.50	82.77	84.75
**Information Gain ratio**	82.72	83.52	78.33	78.35	81.13	84.75
**Gini Index**	82.72	81.52	82.32	80.70	82.77	83.53
**Chi Squared**	82.73	83.12	81.13	82.33	83.17	85.15
**Correlation**	83.13	81.12	81.52	82.70	81.97	83.55
**Relief**	83.95	78.33	81.95	81.88	80.37	85.17
**Uncertainty**	79.50	81.93	80.72	82.73	82.77	84.35
**All features**	79.15	80.70	66.27	74.65	80.77	83.93

**Table 5:** Performance of the prediction algorithms based on the best feature selection approach.

Algorithm	Accuracy (%)	Kappa	Sensitivity (%)	Specificity (%)	PPV (%)	NPV (%)	AUC	Feature selection algorithm
**RF**	84.33	0.605	64.10	93.57	81.97	85.11	0.87	Information Gain
**DT**	83.52	0.581	61.54	93.57	81.36	84.21	0.78	Information Gain ratio
**LR**	82.32	0.590	73.08	86.55	71.25	87.57	0.86	Gini Index
**Naive Bayes**	82.70	0.629	82.05	83.04	68.82	91.03	0.87	Correlation
**W-Bayes Net**	83.17	0.602	73.08	87.72	73.08	87.72	0.91	Chi Squared
**GBT**	**85.17**	**0.644**	**71.97**	**91.23**	**78.87**	**87.64**	**0.93**	**Relief**

Columns PPV and NPV indicate Positive predictive value and Negative predictive value, respectively.

**Fig 3:**
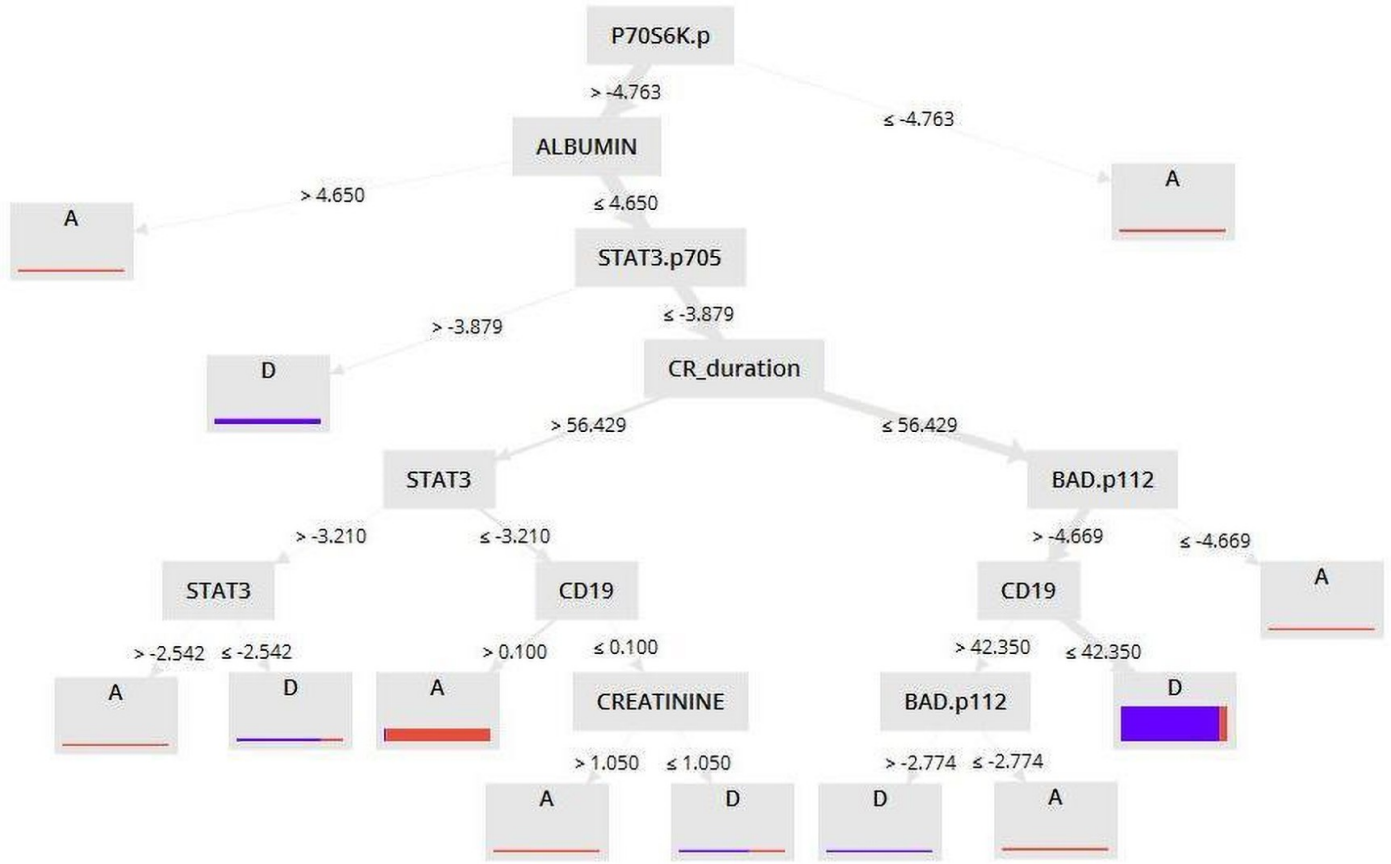
The decision tree pattern performed by RF model with Information Gain dataset.

In the RF method, the dataset that came from the information gain criterion outperform others with an accuracy of 84.33% and AUC of 0.874. Fig 3 shows the decision tree pattern performed by the RF model with the Information Gain dataset. In the DT model, two datasets Information Gain ratio and Chi-Squared achieved close accuracy rates of 83.52% and 83.12%, while they have a precision rate of 81.36% and 76.47%, respectively. Results show that the LR method by Information Gain and Gini Index criteria has a close accuracy rate of approximately 82%, while Information Gain gives a higher precision rate with a precision of 71.79%. Naive Bayes produces approximately the same accuracy with the dataset that comes from the Chi-Squared, Correlation, and Uncertainty criteria (82.33%, 82.70%, and 82.73%, respectively). The W-Bayes Net method produces a higher accuracy and the precision rate with the dataset comes from Chi-Squared criteria (83.17%, 73.08%, respectively). Based on the result of the GBT algorithm, the accuracy of the Relief and Chi-Squared is approximately the same (about 85.17% versus 85.15%), while the Relief criterion gives a higher precision (78.87 and 78.08, respectively). Also, the GBT model with Relief dataset has the Kappa (0.644), Sensitivity (71.97), Specificity (91.23), PPV (78.87), NPV (87.64) and, AUC (0.930). More details are given in S2 Table.

On the other hand, Table 4 shows that the prediction based on all features provides the lowest accuracy rates for the RF, LR, and Naive Bayes models. So, the feature selection datasets produce better accuracy among all feature datasets. Overall, RF and GBT models outperform others with accuracy 84.33% and 85.17%, respectively, while the RF has higher precision (81.97%) and the GBT has a higher AUC (0.930) (Table 5). Considering both precision and sensitivity is useful because there is an imbalance in the observations between the two classes alive and dead.

**Fig 4:**
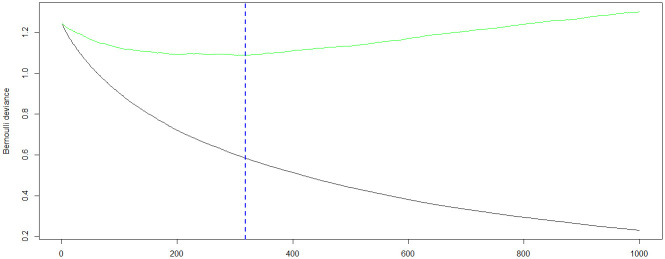
The optimal number of trees in the GBT model.

**Fig 5:**
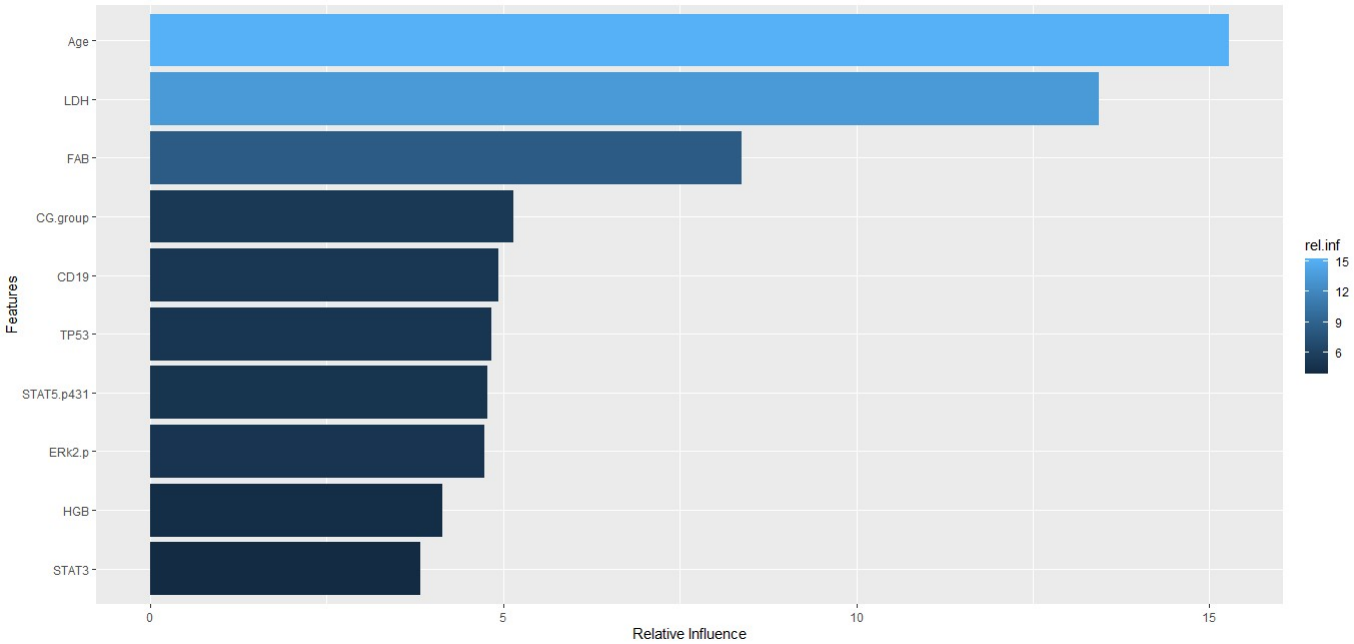
Plot feature importance with top 10 features in the final model GBT.

Due to the CR_duration variable may not be available at disease onset here we carry out prognosis prediction methods again without this feature. Table 6 presents the accuracy (in percentages %) of the prediction algorithms using a 10-fold cross-validation procedure for each of the datasets. As it is shown in the table, the accuracy decreases after removing the CR_duration variable from the model. Also, here again the GBT algorithm produced better accuracy in comparison to other algorithms in model evaluation but on the Information Gain dataset. Now, we give a summary of the GBT model output and produce both a variable importance table and a plot of the model. Fig 4 shows the model deviance as a function of number of trees (N: the number of gradient boosting iteration) using cross validation that is shown with the green color graph in the figure. As can be seen, model deviance increases after a certain number of trees. The value of N that minimizes the deviance is used for the optimal number of trees. Here the optimal number of trees is 318. Fig 5 provides a visual representation of the important features in the final GBT model. Also, Table 7 reports the rank of each feature in the GBT model based on their relative influence, which is a measure indicating the relative importance of each variable in the final model. We can see that Age, LDH, FAB, ERk2.p and, HGB are the five most important variables in our GBT model.

**Table 6:** Accuracy (%) of the prediction algorithms using a 10-fold cross-validation procedure for each of the datasets.

Criterion	RF	DT	LR	Naive Bayes	W-Bayes Net	GBT
**Information Gain**	73.12	71.48	71.08	69.03	73.43	77.55
**Information Gain ratio**	70.27	67.45	63.05	65.08	67.07	69.88
**Gini Index**	72.32	70.30	69.90	69.03	73.43	76.31
**Chi Squared**	71.50	70.33	67.85	71.10	73.52	75.50
**Correlation**	70.28	77.08	71.48	68.23	71.48	77.51
**Relief**	71.90	71.12	72.25	73.10	71.43	74.30
**Uncertainty**	70.68	69.12	67.43	73.90	73.43	77.51
**All features**	68.28	65.87	62.62	64.20	71.83	73.90

**Table 7:** The relative influence each feature in the GBT model.

Features	relative influence
**Age**	15.27358
**LDH**	13.43576
**FAB**	8.380744
**CG.group**	5.140746
**CD19**	4.937505
**TP53**	4.839846
**STAT5.p431**	4.773225
**ERk2.p**	4.72874
**HGB**	4.143627
**STAT3**	3.829885
**P70S6K.p**	3.435698
**ALBUMIN**	3.430047
**BAD.p112**	3.329646
**STAT3.p705**	2.987051
**P70S6K**	2.277646
**ZUBROD.S**	2.07385
**MEK**	1.968749
**BAK**	1.949988
**CREATININE**	1.865403
**SMAC**	1.720244
**MCL1**	1.540197
**BCL2**	1.487394
**XIAP**	1.015671
**AHD**	0.926745
**PRIOR_MAL**	0.508015

### Comparing with cytogenetics

The distribution of patients with favorable, intermediate, or unfavorable cytogenetics among the vital status was significantly uneven (*χ*^2^ test = 29.177 on 2 degrees of freedom, P-value<0.0001; Table 8). Patients with favorable and intermediate cytogenetics were significantly overrepresented in Alive and underrepresented in Dead. Patients with unfavorable cytogenetics were overrepresented in Dead and underrepresented in Alive.

**Table 8:** Distribution of cytogenetic risk groups across the vital status.

			cytogenetic groups		
			Favorable	Intermediate	Unfavorable
Vital Status	Alive	Observed (expected) count	16(6.6)	41(36)	21(35.4)
	Dead	Observed (expected) count	5(14.4)	74(79)	92(77.6)

The three major cytogenetic risk groups are unevenly distributed across the three age groups (*χ*^2^ test = 24.05 on 4 degrees of freedom, P-value<0.0001; Table 9). The percentage of patients with cytogenetics type favorable dropped from 85.7% in those younger than age 56 to 9.5% in those between 56 and 75 years old. Whereas, the proportion of patients with intermediate (or unfavorable) cytogenetics increased from 35.7% (30.1%) in those younger than age 56 to 45.2% (46%) in patients between 56 and 75 years old.

**Table 9:** Distribution of cytogenetic risk groups across the three age groups.

			cytogenetic groups		
			Favorable	Intermediate	Unfavorable
Age group	<56	Observed (expected) count	18(7.8)	41(43)	34(42.2)
	>56 & <75	Observed (expected) count	2(8.9)	52(49)	52(48.1)
	>75	Observed (expected) count	1(4.2)	22(115)	27(113)

## Discussion

In the present study, several machine learning methods were used and compared to predict survival outcomes in patients with AML. Six data mining algorithms were employed, including RF, DT, LR, Naive Bayes, W-Bayes Net, GBT. Based on the obtained accuracy measures, it was shown that all the classification methods performed almost similarly in classifying AML survival with a range between 66.27% and 85.17%. The GBT method produced slightly better accuracy (77.55%), in comparison to other methods in model evaluation. In addition, all classification methods were efficient in predicting the classes for AML survival status.

Previous studies have used prediction models for cancer survivability. For example, Delen et al. used the ANN, DT, and LR methods for the prediction of survival in breast cancer [8]. They showed that DT was the best predictor with 93.6% accuracy, while ANN and LR had 91.2% and 89.2% accuracy, respectively. Similarly, Delen et al. used SVM, DT, ANN, and LR algorithms for the prediction of survival in prostate cancer, in which SVM yielded the best accuracy (92.85%) [11]. Noohi et al. in the evaluation to predict survival of the colon cancer patient, showing that ANN was the most accurate model [34].

Although, most clinical studies reported that RF was the preferred algorithm [12, 13]. Ganggayah et al. showed that the RF algorithm produced slightly better accuracy (82.7%), in comparison to other evaluated algorithms in predicting factors for survival of breast cancer patients [35]. Chebouba et al. proposed to use a stochastic local search meta-heuristic as a feature selection method combined with a random forest classifier to classify AML patients’ response to treatment [36]. They used BAC and the AUC scores as evaluation criteria. They used three types of data: only clinical data, only proteomics data, and finally clinical and proteomics data combined. The numerical results showed that the highest scores are obtained by using clinical data alone, and the lowest score is obtained when using proteomics data alone. Further, their method succeeds in finding promising results compared to the methods presented in the DREAM challenge. Wang et al. proposed the Bayesian nonparametric variable selection approach to identify the prognostic genes for the clinical prognosis prediction of AML [37]. In their study, the overall survival times of AML have been dichotomized with a one-year cutoff and the patients were classified into two subcategories of high-risk and low-risk. There were 53 candidate genes identified from 14,892 genes. With the same parameters and the iteration times in the previous procedures, they separately identified 12, 16, and 18 prognostic genes as features for model construction. The top 18 genes were AADACL1, ABCB10, AIM1, APP, ATF3, BNIP3, DAPK1, DYM, FHL1, GMPR, GUCY1A3, LEF1, MKRN1, MXRA7, NPAL3, SOCS2, TESC, and TM4SF1.

Ganggayah et al. obtained an accuracy between 79.8 to 82.7% in predicting factors for survival of breast cancer patients. The factors that were important in their study were cancer stage, tumor size, number of total axillary lymph nodes removed, number of positive lymph nodes, types of primary treatment, and methods of diagnosis [35].

Based on the results of analysis shown in Tables 4 and 5 among the datasets driven from feature selection, GBT on the Relief dataset has the better performance compared to other algorithms and datasets (with an accuracy of 85.17% and AUC of 0.930). The features in this dataset are CR_duration, CG.group, Age, TP.AFTER.SAMPLE_DATE, CD19, SEX, RACE, PRIOR_MAL, PRIOR_XRT, FIBRINOGEN, PRIOR_CHEMO, PKCA.p, ALBUMIN, CD34, HGB, PTEN.p, FAB, PKCA, STAT5.p431, ITD, LDH, INFECTION, ZUBROD.S, P70S6K, PB_Blast. So, these features could be a better predictor for AML survival in this analysis.

Similar to our findings, Walter et al. declared albumin as one of the factors to determine the risk of treatment-related mortality (TRM) in AML patients by multivariate models [38]. Jabbour et al. using multivariate analysis showed low albumin is an independent adverse factor for CR in AML patients [39]. Also, Liu et al. showed that in the gastric cancer patients’ poor survival was observed with lower levels of BMI (P = 0.028), albumin (P = 0.004), and triglyceride (P = 0.043), respectively. Based on the ROC curve analyses they have suggested that BMI, albumin and, triglyceride have survival-predictor powers similar to the TNM staging system [40]. Other studies showed an inverse association between blood levels of albumin and mortality in the general population [41–45].

Our results highlighted CD19 as one of the main deterministic factors for survival outcomes. CD19 is a cell-surface marker for the diagnosis of B-lineage of mixed-phenotype acute leukemia (MPAL) [4]. Wang et al. used multivariate analysis of clinical features of 188 patients with AML-M2 and showed that CD19 expression is one of the main factors impacting the prognosis of patients. Therefore, it appears that the level of CD19 might be a useful indicator of survival rate. CD19 expression is regulated by paired box transcription factor 5 (PAX5) [46]. Tiacci et al. reported that in most cases of T-cell acute lymphoblastic anemia and AML, PAX5 was not expressed, while PAX5 was expressed in RUNX1-RUNX1T1–positive AML cases (15 of 42 (35.7%) AML cases with RUNX1- RUNX1T1). So the PAX5 might be exceptionally expressed in RUNX1-RUNX1T1–positive AML, and result in CD19 surface expression [47]. Inappropriate PAX5 expression and simultaneously CD19 expression in RUNX1- RUNX1T1–positive AML cases induced bi-phenotypic features and blocked myeloid differentiation [48, 49].

The level of HGB could be another factor in determining survival as our results showed. There is a report that AML patients with monosomal karyotype were associated with significantly older, and lower HGB concentrations and lower WBC counts [50]. Xu et al. reported that low levels of hemoglobin, albumin, lymphocyte, and platelet could serve as a significant risk factor for recurrence-free survival and overall survival in patients with resected pancreatic cancer [51]. They showed that a low level of hemoglobin, albumin, lymphocyte, and platelet was associated with lymph node metastasis, poor tumor differentiation and, high TNM staging [51]. Also, Sweiss et al. suggested that hemoglobin and creatinine clearance are important predictors of outcomes treatment-free survival after autologous stem cell transplantation for multiple myeloma [52]. They reported that lower hemoglobin, lower creatinine clearance, and a combined low hemoglobin and creatinine clearance were strongly associated with improved treatment-free survival [52].

Furthermore, Zhang et al. have found that females with HGB≥100 g/L, FLT3-ITD mutation-negative, and 10 mg/m^2^ Ida were favorable factors for CR [53]. Our results are also shown an association between CR and survival rate. Most patients with newly diagnosed AML achieve CR with induction chemotherapy. Although the majority of patients relapse, despite intensive consolidation chemotherapy. The prognostic factor predicting the duration of the second CR is the duration of the first CR [54]. Ferrara et al. declared that the duration of first CR and cytogenetics are the most applicable prognostic factors in relapsed AML [55].

Similar to what we found regarding the most common protein features of AML patients, Ruvolo et al. showed that p-GSK3α/β, as an indicator of AKT activation, positively correlated with phosphorylation of AKT, BAD, and P70S6K [56]. They suggested AKT-mediated phosphorylation of GSK3α/β as a determinant of the overall survival of AML patients.

Age is generally the most important prognostic factor in AML [57]. Utkarsh et al, (2018) confirmed that aging confers inferior survival in AML, so with every 5-year increase in age hazard ratio rise to 22%. This may be the result of poor performance status, multi-drug resistance, and complicated disease biology, which follow the disease with increasing age [58–60].

In comparison AML patients younger than age 56 with patients older than 75, Multidrug resistance found 33% compared to 57% respectively. Also, the percentage of patients with cytogenetics type favorable dropped from 17% to 4% respectively. Whereas, the proportion of patients with unfavorable cytogenetics increased from 35% to 51% respectively. Also, obvious increases in abnormalities of chromosomes 5, 7, and 17 among observed in the elderly. In the cytogenetic risk group, the increased incidence of unfavorable cytogenetics contributed to their poorer outcome and therapy outcome decline markedly with age [59]. Similar to these studies, as shown in Table 7, in the GBT model the features Age, LDH and FAB were the most influenced features in the analysis. Table 8 showed that proportion of favorable and unfavorable cytogenetic groups significantly different between two vital statuses, and affect the survival of patients. Also, Table 9 showed that with increasing age the proportion of patients with favorable cytogenetics decreased and instead the proportion of unfavorable cytogenetics increased. So the CG.group feature that shows the Cytogenetic classification in AML patients was one of the most important features in the Relief dataset and also influenced features in the GBT model (Tables 3 and 7).

Several studies used the Cytogenetic category for the prediction of survival AML patients [61–63]. Cytogenetic analysis is an important value in the clinical management of patients with AML. Many kinds of chromosome changes may occur in AML cells of patients, some of that can affect a person’s prognosis. Cytogenetic abnormalities most commonly associated with AML have been characterized at the molecular level, also the identification of recurrent chromosomal translocations and inversions associated with this disease prepare the molecular characterization of the chromosomal breakpoint regions [64].

Manola reported that cytogenetic and molecular abnormalities are involved in the pathogenesis of childhood AML, with clonal chromosome abnormalities in 70–85% of cases. He suggested that cytogenetic analysis should be performed in all patients with pediatric AML at diagnosis and during the progress of the disease as additional chromosome abnormalities could predict an imminent relapse. Patients may be stratified to different therapies based on results of conventional cytogenetic analysis, molecular cytogenetic analysis, and/or molecular genetic investigations [65].

Also, Grimwade et al. showed the importance of diagnostic cytogenetics as an independent prognostic factor in AML that providing the framework for a stratified treatment approach of disease [66]. Also, Fröhling et al. reported the clinical importance of cytogenetics and age in patients older than 60 years with AML. They showed that a large subgroup of patients, characterized by age 70 or older or high-risk cytogenetics, or both, had very unfavorable long-term outcomes, with patients above age 70 with high-risk cytogenetics showing a particularly poor prognosis [67].

LDH feature was another important feature in the Relief dataset model (Tables 3 and 7). In Myelodysplastic syndromes (MDS) various mechanisms may cause an increase in LDH. One may be the increased turnover and degradation of myeloid cells in the bone marrow, spleen, and other tissues. Another reason may be ineffective hematopoiesis. Additional cofactors may be an infiltration of the liver and spleen by immature myeloid cells or iron overload. The exact biochemical basis of an increasing LDH in these patients remains unknown [68].

Carobbio et al. reported that LDH values of 40% of AML patients were >1.5 times the upper limit of normal value [69]. Also, Aul et al. have shown that an LDH level of >200 U/l indicates a significantly shorter survival when compared to a lower LDH level (≤200 U/l) [70]. Wimazal et al. showed that an increased LDH level (≥300) in myelodysplastic syndromes is associated with a reduced survival as well as an increased risk to transform to secondary AML [68]. The LDH may therefore be considered as an additional useful parameter in MDS. Wimazal et al. showed that an increase in LDH over time is associated with a higher probability of AML progress and a reduced probability of survival. They recommend using LDH as a prognostic follow-up parameter in MDS [71].

Another feature that was important in the Relief dataset was FAB classification (Tables 3 and 7). Canaani et al. suggested that FAB classification contributes and enhances the prognostic capacity of molecular data provided by FLT3-ITD and NPM1 mutational status. Also, they supported that using FAB classification along with molecular data in AML patients undergoing allogeneic stem cell transplantation [72].

## Conclusion

Rapid prediction of survival of patients at the beginning of treatment would be beneficial for the selection of a better strategy for them and to change it as soon as conditions get worse. The methods used in this study introduced a methodology for analyzing cancer data and other diseases, we applied a combination of feature weighting and machine learning techniques to determine the survival chance of patients. The results of the present study indicated that the 5 top important features that could be used as a predictive measure for the AML patient’s survival include Age, LDH, FAB, Cytogenetic classification, CD19. We have also found that protein features including Albumin, HGB, TP53, STAT3, STAT5.p431, BAD.p112, ERk2.p and, P70S6K.p might be used to predict survival rate in AML patients.

## Supporting information

S1 Table Complete list of protein features in the present study. (DOCX)

S2 Table The Kappa value, sensitivity, specificity, PPV, NPV and AUC of the prediction algorithms using a 10-fold cross-validation procedure for each of the datasets. (DOCX)
