# Supplementary material for: Survival prognostic factors in patients with acute myeloid leukemia using machine learning techniques
Source: PLoS One. 2021 Jul 21;16(7):e0254976. doi: 10.1371/journal.pone.0254976 (PMC8294525; doi:10.1371/journal.pone.0254976)
Supplement: S1 Table — (DOCX) [file pone.0254976.s001.docx]

S1 Table: Complete list of protein features in the present study

| ACTB-AKT-AKT.p308-AKT.p473-BAD-BAD.p112-BAD.p136-BAD.p155-BAK-BAX-BCAT-BCL2-BCLXL-CCND1-DJI-ERK2-ERk2.p-GSK3-GSK3.p-MCL1-MEK-MEK.p-MTOR-MTOR.p-MYC-NRP1-P70S6K-P70S6K.p-PKCA-PKCA.p-PTEN-PTEN.p-S6-S6RP.p240.244-S6.p235-SMAC-SRC-SRC.p527-SSBP2-SSBP3-STAT1.p-STAT3-STAT3.p705-STAT3.p727-STAT5.p431-STAT6.p-SURVIVIN-TP27-TP38.p-TP53-XIAP- HGB- ALBUMIN |
| --- |
