# Supplementary material for: Survival prognostic factors in patients with acute myeloid leukemia using machine learning techniques
Source: PLoS One. 2021 Jul 21;16(7):e0254976. doi: 10.1371/journal.pone.0254976 (PMC8294525; doi:10.1371/journal.pone.0254976)
Supplement: S2 Table — (DOCX) [file pone.0254976.s002.docx]

S2 Tables:

In all bellow tables, columns PPV, NPV indicate Positive predictive value and Negative predictive value respectively.

Performance of Random Forest method by 10-foldcross validation on the different dataset.

| Datasets | Accuracy (%) | Kappa | Sensitivity (%) | Specificity (%) | PPV (%) | NPV (%) | AUC |
| --- | --- | --- | --- | --- | --- | --- | --- |
| Information Gain | 84.33 | 0.605 | 64.10 | 93.57 | 81.97 | 85.11 | 0.874 |
| Information Gain ratio | 82.72 | 0.560 | 58.97 | 93.57 | 83.33 | 80.70 | 0.866 |
| Gini Index | 82.72 | 0.566 | 61.54 | 92.40 | 78.69 | 84.04 | 0.852 |
| Chi Squared | 82.73 | 0.569 | 61.54 | 92.40 | 78.69 | 84.04 | 0.870 |
| Correlation | 83.13 | 0.570 | 58.97 | 94.15 | 83.14 | 83.42 | 0.873 |
| Relief | 83.95 | 0.596 | 62.82 | 93.57 | 81.67 | 84.66 | 0.899 |
| Uncertainty | 79.50 | 0.440 | 46.15 | 94.74 | 80.00 | 79.41 | 0.883 |
| All features | 79.15 | 0.437 | 47.44 | 93.57 | 77.08 | 79.60 | 0.812 |

Performance of Decision Tree method by10-foldcross validation on the different dataset.

| Datasets | Accuracy (%) | Kappa | Sensitivity (%) | Specificity (%) | PPV (%) | NPV (%) | AUC |
| --- | --- | --- | --- | --- | --- | --- | --- |
| Information Gain | 81.52 | 0.554 | 67.95 | 87.72 | 71.62 | 85.71 | 0.768 |
| Information Gain ratio | 83.52 | 0.581 | 61.54 | 93.57 | 81.36 | 84.21 | 0.782 |
| Gini Index | 81.52 | 0.554 | 67.95 | 87.72 | 71.62 | 85.71 | 0.765 |
| Chi Squared | 83.12 | 0.586 | 66.67 | 90.64 | 76.47 | 85.64 | 0.754 |
| Correlation | 81.12 | 0.549 | 67.95 | 87.13 | 70.67 | 85.63 | 0.745 |
| Relief | 78.33 | 0.485 | 62.82 | 85.38 | 66.22 | 83.43 | 0.714 |
| Uncertainty | 81.93 | 0.551 | 61.54 | 91.23 | 76.19 | 83.87 | 0.733 |
| All features | 80.70 | 0.519 | 58.97 | 90.64 | 74.19 | 82.89 | 0.740 |

Performance of Logistic Regression method by10-foldcross validation on the different dataset.

| Datasets | Accuracy (%) | Kappa | Sensitivity (%) | Specificity (%) | PPV (%) | NPV (%) | AUC |
| --- | --- | --- | --- | --- | --- | --- | --- |
| Information Gain | 82.30 | 0.589 | 71.79 | 87.13 | 71.79 | 87.13 | 0.853 |
| Information Gain ratio | 78.33 | 0.457 | 53.85 | 89.47 | 70.00 | 80.95 | 0.803 |
| Gini Index | 82.32 | 0.590 | 73.08 | 86.55 | 71.25 | 87.57 | 0.862 |
| Chi Squared | 81.13 | 0.551 | 66.67 | 87.72 | 71.23 | 85.23 | 0.856 |
| Correlation | 81.52 | 0.566 | 67.95 | 87.72 | 71.62 | 85.71 | 0.853 |
| Relief | 81.95 | 0.564 | 64.10 | 90.06 | 74.63 | 84.62 | 0.861 |
| Uncertainty | 80.72 | 0.545 | 65.38 | 87.72 | 70.83 | 84.75 | 0.861 |
| All features | 66.27 | 0.271 | 58.97 | 69.59 | 46.94 | 78.81 | 0.642 |

Performance of Naive Bayes method by10-foldcross validation on the different dataset.

| Datasets | Accuracy (%) | Kappa | Sensitivity (%) | Specificity (%) | PPV (%) | NPV (%) | AUC |
| --- | --- | --- | --- | --- | --- | --- | --- |
| Information Gain | 81.50 | 0.606 | 82.05 | 81.29 | 66.67 | 90.85 | 0.868 |
| Information Gain ratio | 78.35 | 0.462 | 56.41 | 88.30 | 68.75 | 81.62 | 0.811 |
| Gini Index | 80.70 | 0.591 | 82.05 | 80.12 | 65.31 | 90.73 | 0.866 |
| Chi Squared | 82.33 | 0.596 | 74.36 | 85.96 | 70.73 | 88.02 | 0.872 |
| Correlation | 82.70 | 0.629 | 82.05 | 83.04 | 68.82 | 91.03 | 0.868 |
| Relief | 81.88 | 0.571 | 67.95 | 88.30 | 72.60 | 85.80 | 0.895 |
| Uncertainty | 82.73 | 0.598 | 73.08 | 87.13 | 72.15 | 87.65 | 0.869 |
| All features | 74.65 | 0.451 | 70.51 | 76.61 | 57.89 | 85.06 | 0.797 |

Performance of W-Bayes Net method by10-foldcross validation on the different dataset.

| Datasets | Accuracy (%) | Kappa | Sensitivity (%) | Specificity (%) | PPV (%) | NPV (%) | AUC |
| --- | --- | --- | --- | --- | --- | --- | --- |
| Information Gain | 82.77 | 0.595 | 73.08 | 87.13 | 72.15 | 87.65 | 0.911 |
| Information Gain ratio | 81.13 | 0.543 | 64.10 | 88.89 | 72.46 | 84.44 | 0.877 |
| Gini Index | 82.77 | 0.592 | 73.08 | 87.13 | 72.15 | 87.65 | 0.910 |
| Chi Squared | 83.17 | 0.602 | 73.08 | 87.72 | 73.08 | 87.72 | 0.910 |
| Correlation | 81.97 | 0.583 | 71.79 | 86.55 | 70.89 | 87.06 | 0.906 |
| Relief | 80.37 | 0.538 | 67.95 | 85.96 | 68.83 | 85.47 | 0.902 |
| Uncertainty | 82.77 | 0.592 | 73.08 | 87.13 | 72.15 | 87.65 | 0.911 |
| All features | 80.77 | 0.547 | 69.23 | 85.96 | 69.23 | 85.96 | 0.902 |

Performance of Gradient Boosted Tree method by10-foldcross validation on the different dataset.

| Datasets | Accuracy (%) | Kappa | Sensitivity (%) | Specificity (%) | PPV (%) | NPV (%) | AUC |
| --- | --- | --- | --- | --- | --- | --- | --- |
| Information Gain | 84.75 | 0.630 | 69.23 | 91.81 | 79.41 | 86.74 | 0.927 |
| Information Gain ratio | 84.75 | 0.638 | 71.79 | 90.64 | 77.78 | 87.57 | 0.914 |
| Gini Index | 83.53 | 0.595 | 66.67 | 91.23 | 77.61 | 85.71 | 0.931 |
| Chi Squared | 85.15 | 0.644 | 73.08 | 90.64 | 78.08 | 88.07 | 0.923 |
| Correlation | 83.55 | 0.598 | 67.95 | 90.64 | 76.81 | 86.11 | 0.915 |
| Relief | 85.17 | 0.644 | 71.97 | 91.23 | 78.87 | 87.64 | 0.930 |
| Uncertainty | 84.35 | 0.620 | 69.23 | 91.23 | 78.26 | 86.67 | 0.928 |
| All features | 83.93 | 0.607 | 66.67 | 91.81 | 78.79 | 85.79 | 0.927 |
